# Resilience in Young and Older Adults: Eudaimonic Well-Being Despite Life Challenges

**DOI:** 10.1093/geroni/igaa057.1366

**Published:** 2020-12-16

**Authors:** Shubam Sharma, Susan Bluck, Hsiao-Wen Liao

**Affiliations:** 1 University of Florida, Gainesville, Florida, United States; 2 Stanford University, Palo Alto, California, United States

## Abstract

Although the types of events that occur across life phases may vary, experiencing multiple life challenges in a short time-frame can disrupt mental health (Zepinic, 2016). Maintaining self-continuity (i.e., sense of being the same person over time) when experiencing challenges may, however, foster resilience (Masten, 2001). This study investigated, in both young and older adults, whether: (1) experiencing multiple recent negative life challenges relates to lower current eudaimonic wellbeing, and (2) self-continuity acts as an internal resource for resilience. That is, whether having greater self-continuity ameliorates the negative association between frequent life challenges and well-being. Participants (N = 99 young, 87 older adults) reported all challenging events experienced in the last six years (Sarason et al., 1978). They also completed measures of eudaimonic Well-being (Ryff, 1989) and Sense of Self-continuity (past six years; e.g., Habermas & Köber, 2015). Older adults reported higher eudaimonic well-being than young adults (p < .001). Young adults reported experiencing more challenges (p < .001). Regardless, for both age groups, more frequently experiencing life challenges was associated with lower eudaimonic well-being (p < .001). Self-continuity mediated the association between more frequent experience of challenge and lower eudaimonic well-being: β = .258, SE = .061, 95% CI [.138, .378] (p < .001). Classic risk models focus on the negative effects of life’s inevitable challenges. Our findings, grounded in a resilient aging framework, support the idea that individuals of any age can use internal resources (i.e., strong sense of self-continuity) to maintain or re-establish well-being.

